# Are Freshwater Snails, *Melanoides* sp. and Invasive *Tarebia granifera* (Gastropoda: Thiaridae) Suitable Intermediate Hosts for *Calicophoron microbothrium* (Trematoda: Paramphistomoidea)? An Experimental Study

**DOI:** 10.3389/fvets.2021.705954

**Published:** 2021-07-21

**Authors:** Mokgadi P. Malatji, Nkululeko Myende, Samson Mukaratirwa

**Affiliations:** ^1^School of Life Science, College of Agriculture, Engineering and Science, University of KwaZulu-Natal, Westville Campus, Durban, South Africa; ^2^Department of Research and Scientific Services, National Zoological Garden, South African National Biodiversity Institute (SANBI), Pretoria, South Africa; ^3^Center for Zoonoses and Tropical Veterinary Medicine, Ross University School of Veterinary Medicine, Basseterre, Saint Kitts and Nevis

**Keywords:** *Tarebia granifera*, *Melanoides* spp., *Melanoides tuberculata*, *Melanoides victoriae*, Thiaridae, *Calicophoron microbothrium*, experimental infectivity

## Abstract

Prosobranch snails and adult Paramphistomoidea flukes were collected from water bodies and cattle abattoir located in Mpumalanga province of South Africa, respectively. The snails were identified based on morphological characters as well as the ITS-2 and 16S markers as *Melanoides* sp. and *Tarebia granifera*, respectively, and the Paramphistomoidea flukes were identified as *Calicophoron microbothrium* using the ITS-1/5.8S/ITS-2 marker. After confirming identification, the snails were bred to first filial generation (F1) under laboratory conditions. Ninety snails were randomly selected from the laboratory-bred F1 snails and 25 *Melanoides* sp. and 20 *T. granifera* were exposed to *C. microbothrium* miracidia, and the same numbers were maintained as non-exposed controls. Results showed that *C. microbothrium* successfully established in *Melanoides* sp. and produced cercariae, and the prepatent period recorded was 21 days. Three snails shed cercariae at day 21 postexposure (PE), and rediae and free cercariae were detected in the soft tissues of one snail on dissection at day 44 PE. The same fluke did not establish in *T. granifera*. *Melanoides* sp. started producing offspring at day 7 PE, and *T. granifera* at day 14 PE. In conclusion, our results showed that *Melanoides* sp. used in this study is a suitable intermediate host for *C. microbothrium* under experimental conditions, and given the wide distribution of this snail species, it is important to determine its role in the natural transmission of other *Calicophoron* species that have been reported in South Africa.

## Background

Amphistomosis is a parasitic infection of domestic and wild ruminants caused by immature amphistomes, also known as stomach flukes and located in the small intestines of susceptible hosts (1, 2). It is the most prevalent snail-borne infection in sub-Saharan Africa (3) caused by digenetic trematodes of the superfamily Paramphistomoidea Fischoeder, 1901 (4). According to Mavenyengwa et al. (2), amphistomosis is characterized by scattered epidemics of acute parasitic gastroenteritis in the definitive hosts and results in loss of production, which is associated with high morbidity and mortality, mainly in young animals.

According to Sey (5), more than 70 amphistomes species have been reported globally; however, differentiating between these species is difficult due to the extreme similarities in their morphological features (6). About 32 of these amphistome species in ruminants belong to the families Paramphistomoidea, Gastrothylacidae, and Stephanopharyngidae, and the majority have been recorded in Africa (7). The superfamily Paramphistomoidea has a cosmopolitan distribution and proven to be the main cause of amphistomosis in eastern and southern Africa (4). However, only 10 amphistome species from the genera *Calicophoron, Paramphistomum, Cotylophoron*, and C*armyerius* are regarded as the most common species infecting domestic ruminants (7, 8). Although various amphistomes species have been documented, often amphistomosis outbreaks are caused by certain species, and out of the many Paramphistomoidea species reported in sub-Sharan Africa (7), *Calicophoron microbothrium* (Fischoeder, 1901) has the widest distribution in sub-Saharan African countries (4, 8). This species is the main cause of acute amphistomosis in young animals, which results in losses among infected animals (7).

Like other snail-borne parasitic disease, the transmission of amphistomes and prevalence of amphistomosis are largely influenced by the abundance of the definitive host and the availability and efficiency of the snail intermediate hosts in transmitting the parasites (4). A wide range of pulmonate freshwater gastropods belonging to the genera *Bulinus* Müller (1781), *Biomphalaria* Preston (1910), and *Ceratophallus* Brown and Mandahl-Barth (1973), and *Galba* Müller (1774) from families Planorbidade (Rafinesque, 1815) and Lymnaeidae (Rafinesque, 1815), have been deemed to be the snail intermediate hosts of amphistomes in Africa (4, 9–13). Current literature show that seven freshwater pulmonate snail species—*Biomphalaria pfeifferi* (Krauss, 1848), *Bulinus forskalii* (Ehrenberg, 1831), *Bulinus globosus* (Morelet, 1866), *Bulinus nasutus* (von Martens, 1879), *Bulinus tropicus* (Krauss, 1848), *Ceratophallus natalensis* (Krauss, 1848), and *Galba truncatula* (Müller, 1774)—have been confirmed as natural intermediate hosts of amphistome species in southern and eastern Africa (4), and species of the genus *Bulinus* contribute more in the transmission of the various amphistomes species (4).

Prosobranch snails such as *Melanoides* species and *Tarebia granifera* (Lamarck, 1816) are tropical and subtropical species native to Southeast Asia, the Mediterranean, Pacific islands, East Africa, and across the Middle East (14–16), and Southeast Asia (17, 18), respectively. In South Africa, two *Melanoides* species have been reported to date, namely, *Melanoides tuberculata* (Müller, 1774) and *Melanoides victoriae* (Dohrn, 1865) (19). According to the authors, *M. tuberculata* is widely distributed in the present-day Sahara (20), including South Africa, based on the National Freshwater Snail Collection records (19). *Melanoides victoriae* however appears to be restricted to Southern Africa (14, 21). Of recent, *T. granifera* has been introduced to Africa and other parts of the world either accidentally through aquarium trades or intentionally for biocontrol of schistosome intermediate hosts (22, 23). According to Appleton et al. (17) and de Kock and Wolmarans (19), these species are known to act as intermediate hosts for several trematodes in their native origin. However, their role in the transmission of snail-borne infections, and for amphistomes in particular, is not known in South Africa, albeit there have been reports of successful experimental infection of *M. tuberculata* by *C. microbothrium* in Zimbabwe (24).

Therefore, in order to fully understand the factors influencing the wide distribution of amphistomes species in southern and eastern Africa (4), it is important to accurately identify the snail intermediate hosts and determine their degree of compatibility to *C. microbothrium*, more especially in alien and/or invasive snail species. This information is critical in designing control measures, particularly where amphistomosis in endemic. Hence, this study aimed at experimentally determining the suitability of two prosobranch snails, *Melanoides* sp. and *T. granifera*, as intermediate hosts of *C. microbothrium*.

## Materials and Methods

### Snail Collection and Breeding of F1 Generation

Sexually mature *Melanoides* sp. and *T. granifera* snails used to breed the first filial generation (F1) snails were collected from various water habitats in Mpumalanga province of South Africa, using metal scoops as described by Coulibaly and Madsen (25). Snails were screened for patent trematode infection through shedding (26). Snails that were not shedding cercariae were bred in 2-L polyvinyl chloride (PVC) containers, with eight snails per container, filled with filtered pond water and allowed to produce an F1 generation. Snails were fed blanched dried lettuce supplemented with commercial tropical fish flakes *ad libitum*. The water was changed twice a week, and room temperature was maintained at 27 ± 1°C.

### Amphistomes Collection, Processing, and Hatching of Eggs

Adult amphistomes were collected from rumen of three naturally infected cattle slaughtered at an abattoir in Mpumalanga province of South Africa, and one portion was preserved in physiological saline and the other in 70% alcohol prior to processing at the laboratory. Amphistomes that were preserved in physiological saline were crushed using pestle and mortar to release eggs. The eggs were recovered by passing the suspension through sieves of descending apertures, and eggs were recovered on a 20-μm sieve. Eggs were washed three times in distilled water and then incubated at 27°C for 14 days. Hatching of the embryonated eggs was stimulated by directly exposing the eggs with artificially illuminated light bulb for an hour (27), and free miracidia were then used for infections. The portion preserved in alcohol were processed and identified according to Eduardo (28).

### Snail and Parasite Identification

Thiaridae snails collected from the field were visually and morphologically identified based on the shell images of the South African specimens as described by Brown (14), Miranda et al. (18), and Appleton (29). Soft tissue of up to four individual snails from each species identified based on shell characters were harvested, and DNA was extracted using the Genomic DNA™ Tissue MiniPrep Kit (Zymo Research Corporation, Irvine, CA, USA) according to the manufacturer's instructions. Amplification of the snail DNA was performed based on the 16S rDNA primers and conditions as provided by Palumbi et al. (30) for *T. granifera*. The 16S primers were not successful for *Melanoides* sp.; therefore, the ITS-2 primers and cycling conditions described by Malatji et al. (31) were used. A separate PCR amplification was performed on six amphistome specimens from a batch collected from three cattle at an abattoir and preserved in 70% alcohol prior to using the parasite to infect the F1 snails, and this was based on the ITS-1/5.8S/ITS-2 region using primers and thermocycling conditions described by Mucheka et al. (32). Amplicons were sent to Inqaba Biotechnical Industries (Pty) Ltd. in Pretoria, South Africa, for Sanger sequencing.

Sequences were assembled and manually edited using the BioEdit v7.2.5 program (33). Sequences were aligned along with the homologous sequences obtained from the GenBank database using MUSCLE multiple alignment option on MEGA 7 (34), and alignments were trimmed to a common length of 373 nucleotides for amphistomes, and 399 and 481 nucleotides for *Melanoides* sp. and *T. granifera*, respectively. jModeltest (35) was used to select the best model test of nucleotide substitution suitable for neighbor-joining, maximum likelihood, and Bayesian inference analyses for the ITS-2, 16S, and ITS-1/5.8S/ITS-2 genes. The following models were selected under the Akaike information criterion: the HKY and HKY+G models (36) for ITS-2 and 16S genes, respectively, and the GTR model for ITS-1/5.8S/ITS-2. Maximum likelihood and neighbor-joining trees were generated using PAUP^*^ 4.0 (37) trees, and the nodal support values were estimated using 1,000 bootstrap replicates. Bayesian inference trees were executed using MrBayes 3.1.2 (38), and the nodal support values were indicated as posterior probabilities.

### Experimental Infection

A total of 90 F1 laboratory-bred *Melanoides* sp. (*n* = 50) and *T. granifera* (*n* = 40) snails, with an average shell height of 11–13 and 6–9 mm, respectively, were randomly selected and used in this study. The snails were divided into two treatment groups per species, 25 infected/25 control for *Melanoides* sp. and 20 infected/20 control for *T. granifera*. The selected snails were then allocated to 12 2-L containers, each holding seven to eight snails and were allowed 48 h to acclimatize to the environment.

Forty-five snails, 25 *Melanoides* sp. and 20 *T. granifera*, were then individually exposed to three *C. microbothrium* miracidia as described by Chingwena et al. (24). After exposure, snails were then returned into their respective 2-L containers and fed blanched dried lettuce supplemented with commercial tropical fish flakes three times a week *ad libitum* throughout the duration of the experiment and kept at the same temperature of 27°C.

### Measurements of Parameters

Snail mortality was recorded daily for the experimental groups for 44 days postexposure (PE) to miracidia. Snails were deemed dead if they did not respond to mechanical stimuli, and those that showed movements were returned back into their respective experimental treatments (39). Since female *Melanoides* sp. and *T. granifera* are ovoviviparous, to evaluate reproduction rate, the number of offspring(s) produced were counted every 2 days, and the offspring were removed from the experimental containers. Numbers of offspring were combined to compose weekly collections, and this was done over the 5-week duration of the experiments.

At 3 weeks (21 days) PE, all infected snails were individually exposed to artificial light for 1 h to stimulate cercariae shedding. Shedding was stimulated weekly until the end of the experiments. On day 44 postinfection, shells of surviving snails in the infected group were cracked using a blunt forceps, and the soft tissue were squashed between two glass slides and observed under a stereo microscope (x40) to determine the presence or absence of larval stages of *C. microbothrium*. Snails were deemed infected with *C. microbothrium* if they contained actively crawling elongate rediae with a muscular pharynx or pigmented cercariae in their tissues as described by Chingwena et al. (24). Kolmogorov–Smirnov test on SPSS 24 was used to check if the data sets were normally distributed, while Mann–Whitney U-test was used to test if there was a difference in the mean number of offspring produced between the control and exposed groups of both Thiaridae snails. Bar graphs were plotted using mean and standard deviations to show the trend in the average weekly offspring production using Microsoft Excel 2016.

## Results

### Molecular Identification

**Figure 1:**
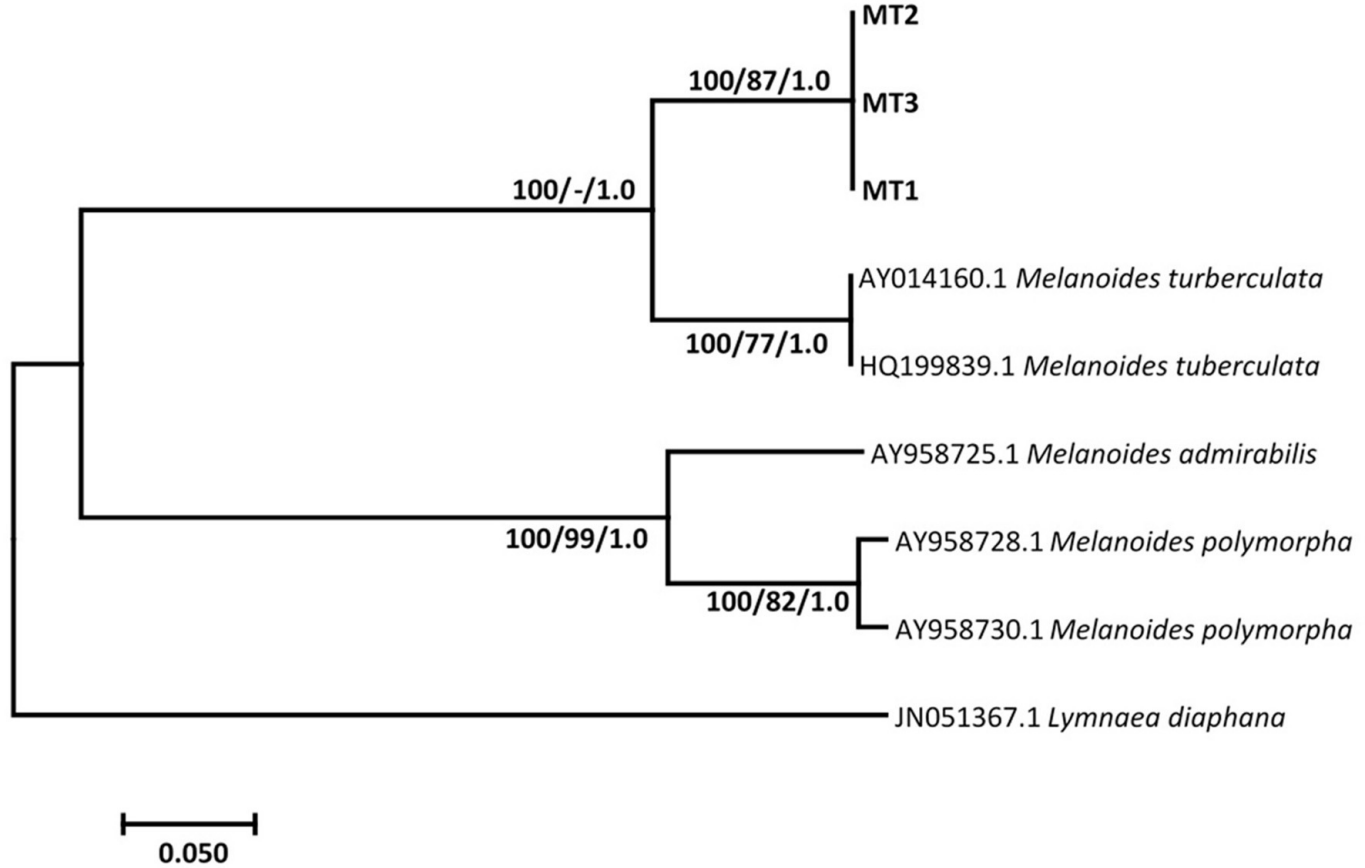
Neighbor-joining tree based on the 399-nucleotide sequence of the ITS-2, confirming the identity of *Melanoides* sp. (MT1-3) (in bold) collected from Mpumalanga province of South Africa and their relationship with other *Melanoides* species obtained from the NCBI GenBank database. The support value indicated on the nodes follows the order neighbor-joining bootstrap value/maximum likelihood bootstrap value/Bayesian inference posterior probability.

**Figure 2:**
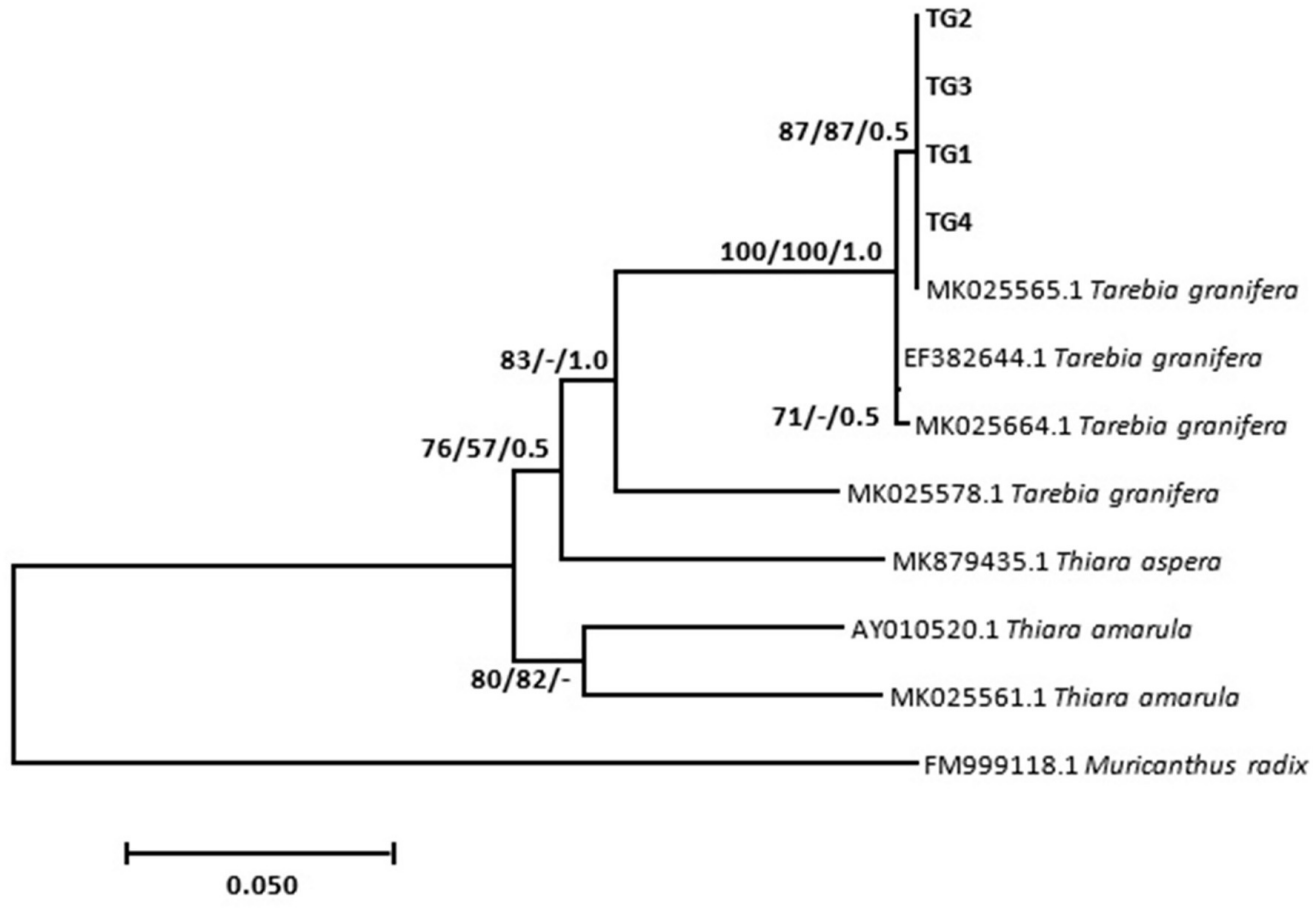
Neighbor-joining tree based on the 481-nucleotide sequence of the 16S rDNA, confirming the identity of *Tarebia granifera* (TG1–4) (in bold) collected from Mpumalanga province of South Africa and their relationship with other Thiaridae species obtained from the NCBI GenBank database. The support value indicated on the nodes follows the order neighbor-joining bootstrap value/maximum likelihood bootstrap value/Bayesian inference posterior probability.

Results show that specimens presumed morphologically to be *M. tuberculata* had homogeneous sequences, which gave a homology of 83.45% with *M. tuberculata* isolate (HQ199839.1). These sequences were deposited in GenBank under the accession numbers MZ229766–MZ229768. Phylogenetic analysis showed that these isolates formed a strong-supported monophyletic sister clade with the clade consisting of GenBank *M. tuberculata* (AY014160.1 and HQ199839.1) (Figure 1). Results also showed that all selected *Tarebia* specimens were confirmed as *T. granifera*. BLAST analysis showed that the specimens gave a 100% homology with *T. granifera* isolate (MK025565.1), and further formed a moderately supported with this GenBank isolate (Figure 2). These specimen's sequences were deposited in GenBank under the accession numbers MZ312250–MZ312253.

**Figure 3:**
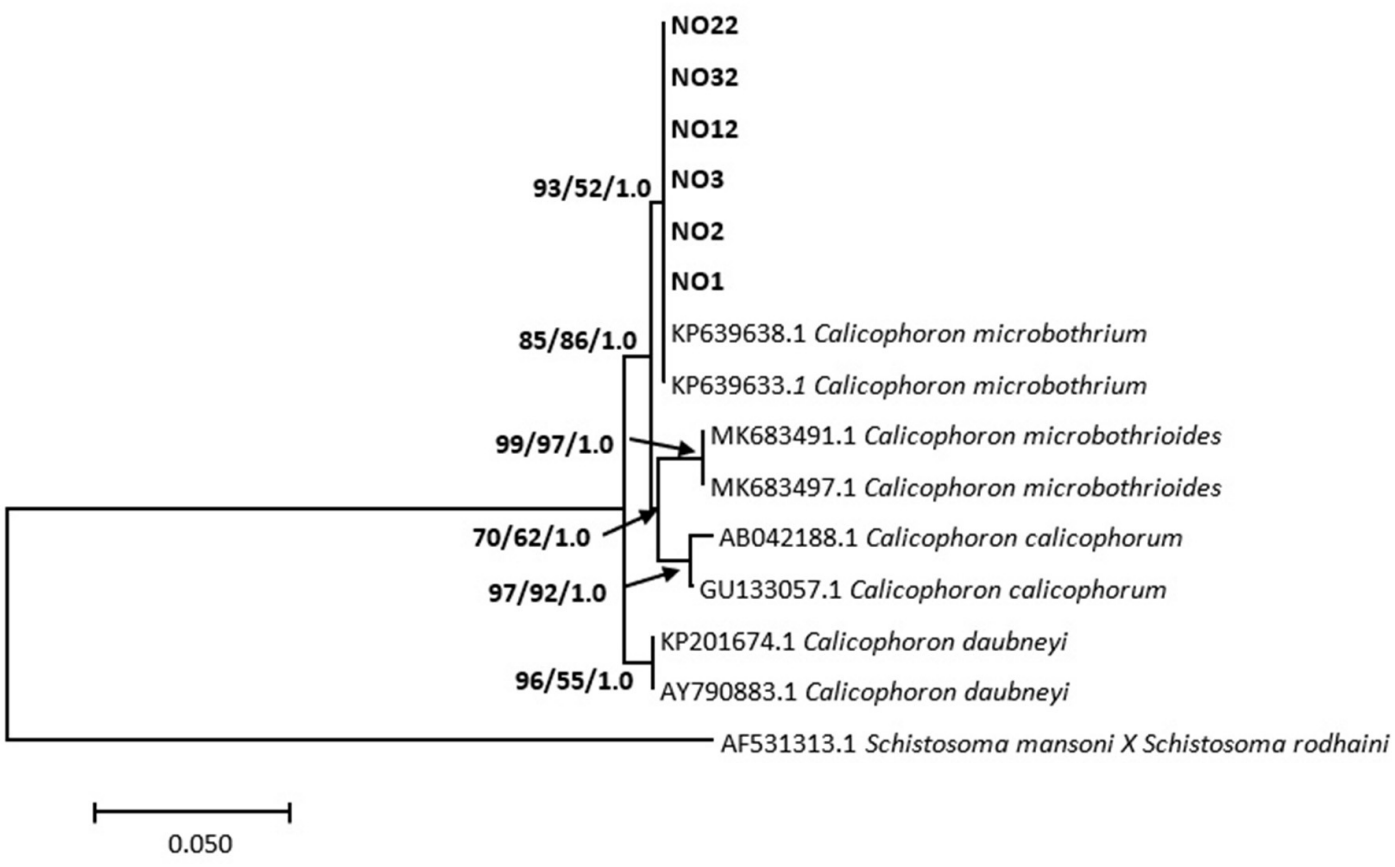
Neighbor-joining tree based on the 373-nucleotide sequence of the ITS-1/5.8S/ITS-2 marker, confirming the identity of amphistomes from Mpumalanga province (in bold) as *Calicophoron microbothrium* and their relationship with *Calicophoron* species obtained from the NCBI GenBank database. The support value indicated on the nodes follows the order neighbor-joining bootstrap value/maximum likelihood bootstrap value/Bayesian inference posterior probability.

Phylogenetic analysis showed the relationship between the *Calicophoron* species as presented in Figure 3. Molecular analysis confirmed the identification of the six analyzed specimens (GenBank accession numbers MZ229627–MZ229632) as *C. microbothrium*, with a homology ranging from 99.25 to 99.50% (*C. microbothrium*, KP639638.1). These specimens formed a novel well-supported clade by neighbor joining with the GenBank *C. microbothrium* isolates (KP639633.1 and KP639638.1) (Figure 3).

### Experimental Infectivity

Experimental infection results showed that *C. microbothrium* established in 4 of 25 (16%) exposed *Melanoides* sp. (Table 1). However, only three of these snails were shedding cercariae as from day 21 PE until day 44 (PE), and one snail was found with rediae when the experiment was terminated. None of the *T. granifera* snails were shedding cercariae during the experiment, and no larval stages were found in the tissues at day 44 PE. There was no mortality recorded from the experimental groups (Table 1).

**Table 1:** Summary of infection and mortality rates of *Melanoides* sp. and *Tarebia granifera* exposed to *Calicophoron microbothrium* miracidia.

**Snail species**	**No. exposed**	**Miracidia per snail**	**NS (Day 44 PE)**	**Infectivity**				
				**NSC (Day 21 PE)**	**NWL (44 days PE)**	**Total infected**	**% Infectivity**	**Mortality (%)**
*Melanoides* spp. —exposed group	25	3–5	25	3	1	4	16	0
*Melanoides* spp. —Control	25	N/A	25	N/A	N/A	N/A	N/A	0
*T. granifera—*exposed group	20	3–5	20	0	0	0	0	0
*T. granifera*-control	20	N/A	20	N/A	N/A	N/A	N/A	0

*NS, Number of snails surviving at day 44 post-exposure to miracidia; NSC, Number of snails shedding cercariae at day 21 post-exposure to miracidia; NWL, Number of snails with larvae at day 44 post-exposure to miracidia; N/A, Not applicable*.

Table 2 shows the average number of offspring produced by both the exposed and non-exposed thiarid snails for a period of 44 days. The nonexposed groups (Melanoides sp. = 150, *T. granifera* = 30) generally produced more offspring for both thiarid species over the 44 days of the experiments as compared to the exposed groups (*Melanoides* sp. = 121, *T. granifera* = 8); however, the differences were only statistically significant (*p* < 0.05) at week 2 for the *Melanoides* sp. groups, and at weeks 4 and 5 for the *T. granifera* groups (*p* < 0.05) (Table 2).

**Table 2:** Weekly average production of offspring by exposed to *Calicophoron microbothrium* miracidia and non-exposed groups of *Melanoides* sp. and *Tarebia granifera*.

	***Melanoides*** **spp**.			***T. granifera***		
**Weeks**	**Non-exposed ($\bar{\text{X}}\;$± SD)**	**Exposed ($\bar{\text{X}}\;$± SD)**	***p*-value**	**Non-exposed ($\bar{\text{X}}\;$± SD)**	**Exposed ($\bar{\text{X}\;}$± SD)**	***P*-value**
1	2.50 ± 2.11	1.33 ± 1.57	0.418	0	0	–
2	1.75 ± 0.87	1.58 ± 1.08	0.001^*^	0.25 ± 0.70	0.13 ± 0.35	0.262
3	2.58 ± 1.17	2.33 ± 1.07	0.420	0.38 ± 0.74	0.13 ± 0.35	0.405
4	2.75 ± 1.06	2.41 ± 1.72	0.425	1.75 ± 1.28	0.38 ± 0.52	0.011^*^
5	2.98 ± 1.24	2.41 ± 0.79	0.123	2.00 ± 1.72	1.18 ± 1.02	0.014^*^

* *Significant difference at P < 0.05*.

## Discussion

Molecular and BLAST analyses based on the ITS-1/5.8S/ITS-2 characterized all six isolates of amphistomes collected from the abattoir for this study as *C. microbothrium*, with a percentage similarity of more than 99% with an isolate from Zimbabwe (KP639638.1). The isolates formed a strongly supported clade with the GenBank *C. microbothrium* sequences and formed a sister clade to the *C. microbothrioides* clade by a bootstrap value of 92% by neighbor joining. Furthermore, there was no sequence divergence and diversity found within the *C. microbothrium* populations from Mpumalanga (South Africa) with GenBank sequences from Zimbabwe (KP639638.1) and South Africa (KP639633.1). Based on the literature (4, 8), *C. microbothrium* is widely distributed in southern Africa, and this species has been implicated in several amphistomosis cases in young ruminants in eastern and southern Africa (4).

Comparing the sequences of Thiaridae species through BLAST, the sequences from this study confirmed the identification of our specimens as *Melanoides* sp. and *T. granifera* with percentage similarities of 83.45 % and 100 % to *Melanoides tuberculata* and *T. granifera*, respectively. The *Melanoides* isolates showed a mean genetic distance of 10.4% from isolates obtained from the GenBank database. This divergence was further illustrated on the phylogenetic tree, where three of the *Melanoides* sp. species formed a strong supported sister clade to the Iranian (HQ199839.1) and United Kingdom (AY014160.1) isolates. *M. tuberculata* and *M. victoriae* are the only two species from the genus that have been reported in South Africa. The absence of *M. victoriae* in the GenBank database meant that we could not have a complete comparison of the sequences we got from our isolates considering that two *Melanoides* species have been documented in South Africa. However, on comparison of shell characters of our experimental specimens with those documented for *M. victoriae* and *M. tuberculata* (14), our specimens were more compatible with *M. tuberculata* rather than *M. victoriae*. As a result, the species we used in our experiments were designated as *Melanoides* sp., and the genetic difference observed could be due existence of clones as some populations have been reported to be entirely female and propagate parthenogentically (14), with a possibility that it could be a mixture of two clones.

The *T. granifera* isolates from this study showed a genetic variation ranging from 0.0 to 0.08% from the reference sequences obtained from GenBank. However, no genetic variation was observed within sequences of our study population. These species formed a strongly supported clade by neighbor joining and Bayesian inference, supporting the identification of our isolates as *T. granifera*. Although *M. tuberculata* is regarded indigenous to South Africa (28, 40), both Thiaridae species are considered invasive in their allochthonous regions (15, 16, 18, 22, 23), and their role in the transmission of snail-borne parasites has not yet been intensively explored in South Africa. Experimental studies have shown that parasites tend to be more infective and increase in virulence and transmission against the native or local hosts (sympatric) as compared to the foreign host (allopatric) population (41). This was observed in this study, where results showed that of the two Thiaridae snail species studied, only *Melanoides* sp. snails were susceptible to *C. microbothrium*. This is not surprising as two *Melanoides* species have been recorded in South Africa to date (19), and of these two species, *M. tuberculata* is not only considered endemic and widespread in South Africa (15, 29) but also proven to be a compatible intermediate host of various snail-borne parasites (19, 27); this includes successful experimental infections of this amphistome species in Zimbabwe (24). The unsuccessful infection in *T. granifera* recorded in this study may possibly be due to failure of *C. microbothrium* to adapt to this invasive freshwater snail (42).

Chingwena et al. (24) reported an infection rate of 5.9% in *M. tuberculata* snails infected with *C. microbothrium*, with a mortality rate of 29.2%. This infection rate is lower than the observed 16% infection recorded in *Melanoides* sp. from this study, with no mortalities. Although the author further concluded that this snail species could potentially act as an intermediate host of *C. microbothrium*, since the infected snails did not shed the cercariae, infection was only confirmed through the observation of the rediae with cercariae upon dissection (24). However, in this study, 12% of the infected *Melanoides* sp. snails started shedding cercariae at day 21 PE until day 44 PE, with only one snail (4%) found with rediae and free cercariae on dissection. The findings from this study and that of Chingwena et al. (24) show that not only can *Melanoides* sp. sustain full development of *C. microbothrium*, but this also demonstrates that this snail species is able to shed cercariae and hence be able to transmit the parasite to the definitive host. However, the magnitude in which *Melanoides* sp. contributes to the transmission of *C. microbothrium* to ruminants under natural conditions still needs to be assessed (24).

According to Brown (14) and Veeravechsukij et al. (43), both *T. granifera* and *M. tuberculata* are ovoviviparous and reproduce parthenogenetically (14, 43), and this might be one of the key characteristic of *T. granifera*'s success as an invader. Results from the present study showed that *Melanoides* sp. experimental groups started producing offspring from the first week of the experiments. However, the number of offspring produced by the control group (non-exposed) significantly declined at week 2 (*p* < 0.05), followed by a gradual increase from week 3 to termination, and this might be due to multiple biological factors. However, *T. granifera* snails only started on week 2 of the experiments, and the number of offspring produced gradually increased up to week 5. The number of offspring produced per week was significantly different (*p* < 0.05) on weeks 4 and 5 for both exposed and control groups. This delay in reproduction is surprising since previous field and experimental studies in Florida and Puerto Rico showed that an estimate sexual maturing of *T. granifera* species takes place when the snail shell height is 5.5–8.0 mm (44) and 6.0–7.0 mm (45). Furthermore, Appleton et al. (17) reported the presence of blasted stage embryos in the brood pouches of snails as small as 8.0 mm. Despite the delay in reproduction observed from this study, sexual maturity started when the shell heights were about 8 mm for the infected and 10 mm for the control, which is in line with other studies (17, 44, 46).

## Conclusion

Results from this study have shown the ability of *Melanoides* sp. to sustain infection with *C. microbothrium* and to shed cercariae as early as day 21 PE. In this study, the snail can act as an invertebrate host *C. microbothrium* under laboratory conditions, but the extent to which it is contributing to natural transmission of this parasite in South Africa and whether the snail is susceptible to other amphistome species from the genus *Calicophoron* is unknown. Furthermore, this study has also confirmed that the strain of *T. granifera* used in this study is refractory to infection with *C. microbothrium* under laboratory conditions. Future research efforts should focus on collecting other invasive freshwater snail species and assessing trematode infections using combined techniques such as shedding of cercariae, detection of presence of larval stages in fresh snail tissues, and use of molecular techniques (PCR) to confirm infection as done in this study.

## Data Availability Statement

The original contributions presented in the study are publicly available. This data can be found at: GenBank under the accession numbers MZ229766–MZ229768.

## Ethics Statement

The study was reviewed and approved by the Animal Research Ethics Committee (AREC/041/017M) of the University of KwaZulu-Natal, South Africa.

## Author Contributions

SM and MM conceptualized the study and collected samples. MM and NM performed laboratory work and analyzed the results. MM wrote the first draft of the manuscript. All authors read, edited, and agreed on the final version.

## Conflict of Interest

The authors declare that the research was conducted in the absence of any commercial or financial relationships that could be construed as a potential conflict of interest.
